# A mathematical model for predicting cardiovascular responses at rest and during exercise in demanding environmental conditions

**DOI:** 10.1152/japplphysiol.00619.2021

**Published:** 2022-06-02

**Authors:** Alex Lloyd, Dusan Fiala, Christian Heyde, George Havenith

**Affiliations:** ^1^Environmental Ergonomics Research Centre, Loughborough University, Loughborough, United Kingdom; ^2^ERGONSIM—Human Thermal Modelling, Messstetten, Germany; ^3^Adidas Sport Science Team, Herzogenaurach, Germany

**Keywords:** body temperature, cardiovascular strain, mathematical modeling, simulation, thermoregulation

## Abstract

The present research describes the development and validation of a cardiovascular model (CVR Model) for use in conjunction with advanced thermophysiological models, where usually only a total cardiac output is estimated. The CVR Model detailed herein estimates cardio-dynamic parameters (changes in cardiac output, stroke volume, and heart rate), regional blood flow, and muscle oxygen extraction, in response to rest and physical workloads, across a range of ages and aerobic fitness levels, as well as during exposure to heat, dehydration, and altitude. The model development strategy was to first establish basic resting and exercise predictions for cardio-dynamic parameters in an “ideal” environment (cool, sea level, and hydrated person). This basic model was then advanced for increasing levels of altitude, heat strain, and dehydration, using meta-analysis and reaggregation of published data. Using the estimated altitude- and heat-induced changes in maximum oxygen extraction and maximum cardiac output, the decline in maximum oxygen consumption at high altitude and in the heat was also modeled. A validation of predicted cardiovascular strain using heart rate was conducted using a dataset of 101 heterogeneous individuals (1,371 data points) during rest and exercise in the heat and at altitude, demonstrating that the CVR Model performs well (*R*^2^ = 0.82–0.84) in predicting cardiovascular strain, particularly at a group mean level (*R*^2^ = 0.97). The development of the CVR Model is aimed at providing the Fiala thermal Physiology & Comfort (FPC) Model and other complex thermophysiological models with improved estimations of cardiac strain and exercise tolerance, across a range of individuals during acute exposure to environmental stressors.

**NEW & NOTEWORTHY** The present research promotes the adaption of thermophysiological modeling to the estimation of cardiovascular strain in individuals exercising under acute environmental stress. Integration with advanced models of human thermoregulation opens doors for detailed numerical analysis of athletes’ performance and physiology during exercise, occupational safety, and individual work tolerability. The research provides a simple-to-validate metric of cardiovascular function (heart rate), as well as a method to evaluate key principles influencing exercise- and thermoregulation in humans.

## Graphical abstract

**Figure:**
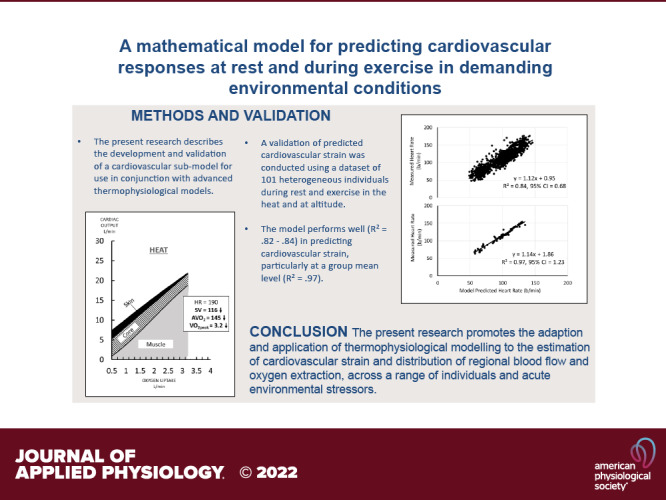

## INTRODUCTION

Multisegmental modeling of human thermoregulation provides an established predictive tool for estimating changes in body temperature, as well as metabolic, vascular, sudomotor, and perceptual responses to thermal stress (1). Based on the immediate environmental parameters surrounding a human, as well as their physical workload and clothing, modeling enables a wide range of key thermophysiological outputs to be computed, including skin wettedness, core temperature, skin temperatures, perspiration rate, thermoregulatory and sensation responses, and environmental heat losses (1, 2). To date, several multisegmental models of human thermoregulation have been proposed (3–7); one of the most recent is the Fiala thermal Physiology & Comfort (FPC) Model (8). Although the current iteration of the FPC and other human thermoregulation models (5, 6, 9) provide valuable predictive insights on body temperatures, fluid loss, and human thermal comfort, they are less well adapted to applications characteristic of intense physical exercise. This is in part due to limited cardiac-specific modeling, where models mainly define cardiac output based on metabolic rate and skin blood flow requirements, but typically do not consider constraints to its control and limitations. For example, the noted models do not utilize stroke volume and heart rate parameters, and therefore do not actively represent the relative levels and limits of cardiovascular strain imposed by passive and exercise heat stress, dehydration, age, aerobic fitness level, or the cardiorespiratory strains imposed by altitude.

The motivation for the present research was to advance the capabilities of the FPC Model and other multisegmental models of human thermoregulation to predict cardiovascular responses and associated constraints related to intense exercise and exposures to challenging environmental conditions. Although the new “Cardiovascular Response Model” (CVR Model) was developed as a separate, stand-alone, predictive tool, the underlying concept facilitates its implementation as a submodel in advanced thermophysiological simulation model networks. Such an incorporation bears prospects of extending the applicability of thermophysiological models to demanding athletic, military, occupational, and public health (e.g., heatwaves) settings, by providing a detailed description of the strains and limitations experienced by humans operating in such conditions. Predictions of thermophysiological body states may then be performed taking into account the upper bounds of cardiovascular factors, including maximum heart rate, maximum cardiac output, or maximum muscle oxygen extraction. This in turn provides a capacity to use thermophysiological models in the identification and prevention of occupational injury or harm in demanding environmental settings. A further benefit of incorporating cardiovascular parameters is the possibility for wide-ranging model performance testing against readily available, field-based validation metrics (such as heart rate), as opposed to limited and more complex laboratory measures, such as body temperature, sweat rates, or cardiac output. This in turn provides an additional means by which thermophysiological models, as well as our basic understanding of exercise- and thermoregulation in humans, can be mathematically validated.

The primary purpose of the present research was to describe a cardiovascular model, i.e., the CVR Model, for the estimation of cardiovascular responses (cardiac output, stroke volume, heart rate, and regional blood flow) to rest and exercise in various environmental conditions. The secondary and tertiary aim of the research was to devise a model that would allow subsequent integration into existing thermophysiological models and finally validate the CVR Model for its capacity to predict heart rate responses in healthy humans.

## MATERIALS AND METHODS

### General Approach to Model Development

The CVR Model estimates cardiovascular responses (e.g., changes in heart rate, stroke volume, cardiac output, and regional blood flow) to physical workloads, as well as by changes in age, aerobic fitness, heat strain, dehydration, and altitude. The model has been developed based on two fundamental physiological equations, i.e., the Fick principle (*Eq. 1*) and the cardiac output principle (*Eq. 2*):

(*1*) $${\dot{\text{V}}\text{O}}_{2}=\text{CO}\cdot {\text{AVO}}_{{\text{2}}_{\text{diff}}}\;\;\;\;\;(\text{L}\cdot {\text{min}}^{-\text{1}})$$

(*2*) $$\text{CO}=\text{SV}\cdot \text{HR}\;\;\;\;\;(\text{L}\cdot {\text{min}}^{-\text{1}})$$

where V̇o_2_ is oxygen consumption (L·min^−1^); CO is cardiac output (L·min^−1^); ${\text{AVO}}_{2_{\text{diff}}}$ the arterial blood oxygen extraction (${\text{L}}_{\left[{\text{O}}_{2}\right]}$·L_[blood]_^−1^); SV stroke volume (L); HR heart rate (beats·min^−1^).

The arterial blood oxygen extraction is defined as the difference between the oxygen content of arterial blood, i.e., pulmonary vein O_2_ (${\text{L}}_{\left[{\text{O}}_{2}\right]}$·L_[blood]_^−1^), and venous blood, i.e., pulmonary artery O_2_ (${\text{L}}_{\left[{\text{O}}_{2}\right]}$·L_[blood]_^−1^), respectively:

(*3*) $${\text{AVO}}_{2_{\text{diff}}}={\text{pulmonary vein O}}_{2}-{\text{ pulmonary artery O}}_{2}\;({\text{L}}_{\left[{\text{O}}_{2}\right]}\cdot {{\text{L}}_{\left[\text{blood}\right]}}^{-\text{1}})$$

Using the aforementioned equations, the general strategy was to first develop a “basic” CVR Model that establishes resting and exercise predictions for cardiac output (CO), stroke volume (SV), and heart rate (HR) parameters in the “ideal” laboratory environment, e.g., neutral temperature, sea-level, and low carbon dioxide conditions. Note that the term “basic” is used throughout this research paper to describe cardiovascular parameters that are not yet modulated for environmental strain (e.g., “basic CO” would be cardiac output not modulated for changes in hydration, body temperature, or suboptimal oxygenation).

Upon establishing the fundamental relationships of the basic CVR Model, changes in both resting and maximum oxygen extraction at the muscle (${\text{AVO}}_{2_{\text{diffrest}}}$ and ${\text{AVO}}_{2_{\text{diffmax}}}$, respectively) due to altitude ascent were incorporated. The necessary increases in CO to compensate changes in oxygen extraction (${\text{AVO}}_{2_{\text{diff}}}$) were then calculated and reintegrated into the CVR model. Next, the model SV, HR, and CO were modulated for increasing levels of heat strain and dehydration, across the full range of exercise intensities, i.e., from resting activity through to maximum oxygen consumption (V̇o_2max_). Using the predicted modulations of maximum SV (SV_max_) and maximum cardiac output (CO_max_) in the heat, as well as reduction in ${\text{AVO}}_{2_{\text{diffmax}}}$ due to altitude, the changes in V̇o_2max_ observed under heated and/or high-altitude conditions were modeled. To complete the CVR Model development, the redistribution of regional blood flows to different tissues was estimated.

For all model developments, the literature was surveyed for appropriate studies that detailed the key variables and relationships necessary for each phase of development. The rationale behind the selection of the literature, and the analyses conducted, are discussed in detail in the following paragraphs. Upon completion of the model development, a validation of the CVR Model was conducted against independent datasets of heterogeneous individuals varied by age, fitness, and body morphology, during rest and exercise in the heat (10, 11) and at altitude (Tsuji et al., 2019, unpublished observations).

#### Defining physical workloads.

In the CVR Model, the workload is defined as fraction of the difference between resting V̇o_2_ (V̇o_2rest_) and 100%, V̇o_2max_, i.e., it is the fraction of V̇o_2max_ reserve (hereafter termed: FV̇o_2max(reserve)_). To calculate V̇o_2rest_, resting energy expenditure was estimated using the Mifflin–St Jour equation, then converted from kcal·day^−1^ to L·min^−1^ by rearranging the Weir formula, assuming a respiratory exchange ratio of 0.7 (12, 13).

(*4.1*) $${\text{Male }\dot{\text{V}}\text{O}}_{\text{rest}}=\frac{10\cdot \text{Mass}+6.25\cdot \text{Height}+5\cdot \text{Age}+5}{1,440\cdot 5.05\cdot \text{RER}}(\text{L}\cdot {\text{min}}^{-\text{1}})$$

(*4.2*) $${\text{Female }\dot{\text{V}}\text{O}}_{\text{rest}}=\frac{10\cdot \text{Mass}+6.25\cdot \text{Height}+5\cdot \text{Age}-161}{1,440\cdot 5.05\cdot \text{RER }}(\text{L}\cdot {\text{min}}^{-\text{1}})$$

(*5*) $${\text{F}\dot{\text{V}}\text{O}}_{2{\text{max}}_{\left(\text{reserve}\right)}}=\frac{{\dot{\text{V}}\text{O}}_{2}-{\dot{\text{V}}\text{O}}_{2\text{ rest}}}{{\dot{\text{V}}\text{O}}_{2\text{max}}-{\dot{\text{V}}\text{O}}_{2\text{ rest}}}\;\left(\text{Fraction}\right)$$

where mass is the pre-exposure mass of the individual (kg); height is the stature of the individual (cm); age is the age of the individual (years); V̇o_2_ is the immediate oxygen consumption at that moment of rest (i.e., female or male V̇o_2rest_) or exercise (L·min^−1^); RER is a respiratory exchange ratio of 0.7; and V̇o_2max_ is the basic maximum oxygen consumption of that individual (L·min^−1^).

### The Basic Model

Although several existing thermoregulatory models contain cardiac output metrics per se, this value is typically derived from the tissue oxygen needs, i.e., metabolic rate, assuming a fixed muscle oxygen extraction of 200 mL O_2_/L of blood (3, 6), and skin blood flow necessary to regulate the body temperature (4). Neither of these values account for cardiac strains imposed by, for example, the heat-induced competitive redistribution of blood flow, or the altitude-induced limitations in oxygen extraction capacity at the muscle. Perhaps more critically, the current thermophysiological models do not typically provide individual CO_max_, SV_max_, and maximum heart rate (HR_max_) values, which are needed to estimate cardiodynamic responses to rest and exercise, and to define cardiac limitations to exercise. Thus, it was first necessary to develop an independent cardiac output model, which represents the cardiac function in response to rest and exercise in moderate temperature, well-ventilated environments. As noted earlier, these parameters are termed “basic,” as they are not yet modulated for environmental strains (e.g., dehydration, heat, and altitude).

#### Resting and maximum stroke volume.

The Fick principle dictates that V̇o_2max_ is a function of ${\text{AVO}}_{{\text{2}}_{\text{diffmax}}}$ and CO_max_ (*Eq. 1*); and that CO_max_ is a function SV_max_ and HR_max_ (*Eq. 2*). It was therefore necessary to first develop algorithms to calculate SV_max_. V̇o_2max_ is a key input variable used in predictive thermophysiological models, due in part to its relative ease of measurement in humans, but also it is widespread reporting across heterogeneous populations. Thus, the relation between SV_max_ (mL·beat^−1^) and absolute V̇o_2max_ (L·min^−1^) was examined, with a view using V̇o_2max_ as a predictor variable for SV_max_. Absolute V̇o_2max_ (i.e., L·min^−1^) was used as CO_max_ (and thereby SV_max_) are dependent on fitness level and body mass, as opposed to a mass normalized fitness level per se.

**Figure 1.**
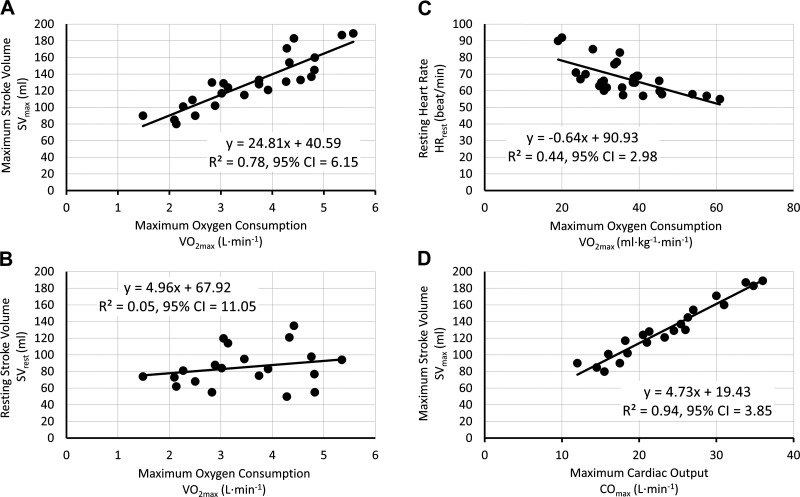
Basic model development. *A*: correlation between maximum stroke volume (mL) and maximum oxygen consumption (L·min^−1^) across nine studies and 25 populations groups (*n* = 251). *B*: correlation between stroke volume at rest (mL) and maximum oxygen consumption (L·min^−1^), across seven studies and 20 populations groups (*n* = 206). *C*: correlation between resting heart rate (beats·min^−1^) and maximum oxygen consumption (mL·kg^−1^·min^−1^), across 12 studies and 28 population samples (*n* = 269). *D*: correlation between maximum stroke volume (mL) and maximum cardiac output (L·min^−1^), across eight studies and 21 populations groups (*n* = 219).

Data for V̇o_2max_ and SV_max_ were aggregated from nine research studies (*n* = 25 population groups, total *n* = 251 participants) spanning a range of ages (35.2 ± 16.7 yr, range 18–81 yr), sexes, fitness levels, and training statuses. Global group mean V̇o_2max_ in these studies was 3.6 ± 1.1 L·min^−1^ (53.5 ± 16.5 mL·kg^−1^·min^−1^), ranging between 1.5 and 5.57 L·min^−1^ (22.2–84.1 mL·kg^−1^·min^−1^) (14–22). Of the 25 groups, SV was calculated using acetylene rebreathing and electrocardiography in 18 groups (16–21), carbon dioxide rebreathing and electrocardiography in six groups (15, 22), with the remaining group estimated using dye dilution and electrocardiography (14). The result of the aggregation was a strong linear relation, indicating that 78% of the variance in SV_max_ is explained by changes in V̇o_2max_ (Fig. 1*A*). Using this relationship, an equation to predict SV_max_ (*Eq. 6*) from measured or estimated V̇o_2max_ was developed.

The same process was then conducted on a reduced data set of 20 population groups (*n* = 206) to calculate resting SV (SV_rest_; Fig. 1*B*). However, no clear relationship was observed (15–21). Given SV_rest_ is imperative to modeling SV across exercise intensities, for this purpose the mean SV value from these studies (i.e., 85.1 mL·beat^−1^) was used (*Eq. 7*).

(*6*) $${\text{SV}}_{\text{max}}=40.59+24.81\cdot {\dot{\text{V}}\text{O}}_{2\text{max }}\;(\text{mL}\cdot {\text{beat}}^{-\text{1}})$$

(*7*) $${\text{SV}}_{\text{rest}}=85.1\;(\text{mL}\cdot {\text{beat}}^{-\text{1}})$$

where V̇o_2max_ is basic maximum oxygen consumption of that individual (L·min^−1^).

#### Resting and maximum heart rate.

Age is a key variable known to impact HR_max_ and equations to calculate changes in HR_max_ with age are well established and widely validated (*Eq. 8*) (23). Aerobic fitness is also generally thought to lower resting heart rate (HR_rest_), through modulations in cardiac parasympathetic and sympathetic activity (24), although this remains equivocal (25). To estimate the effects of aerobic fitness (i.e., V̇o_2max_) on HR_rest_, data from 12 studies were aggregated and re-analyzed, including both cross-sectional and longitudinal HR_rest_ data (*n* = 28 population groups, total *n* = 269 participants) (25–36). These studies also spanned a range of ages (42.7 ± 15.2 yr, range 20–66 yr), sexes, and fitness levels, with a global group mean V̇o_2max_ of 36.2 ± 10.3 mL·kg^−1^·min^−1^ (range: 19.0–60.9 mL·kg^−1^·min^−1^) and a HR_rest_ values of 67 ± 10 beats·min^−1^ (range: 55–92 beats·min^−1^) (25–36).

Contrary to estimated SV_max_, mass normalized V̇o_2max_ (i.e., mL·kg^−1^·min^−1^) provided a better estimated of HR_rest_ than absolute V̇o_2max_. A mild linear relation was observed, indicating that 44% of the variance in HR_rest_ is explained by changes in body mass corrected V̇o_2max_ (Fig. 1*C*; *Eq. 9*). Using these data, the following equations were used to represent HR_rest_ and HR_max_ in the CVR Model.

(*8*) $${\text{HR}}_{\text{max}}=208-0.7\cdot \text{age}\;(\text{beats}\cdot {\text{min}}^{-\text{1}})$$

(*9*) $${\text{HR}}_{\text{rest}}=90.93-0.64\cdot \frac{{\dot{\text{V}}\text{O}}_{2\text{max }}}{\text{mass}}\cdot 1000\;(\text{beats}\cdot {\text{min}}^{-\text{1}})$$

where V̇o_2max_ is baseline maximum oxygen consumption of that individual (L·min^−1^); and age is time passed since birth (years). HR_max_ is taken directly from Tanaka et al. (23).

#### Resting and maximum cardiac output.

Using the basic SV and HR equations described earlier, the CO principle (*Eq. 2*) can be rearranged to calculate resting cardiac output (CO_rest_), CO_max_, and intensity-dependent CO, as detailed as follows:

(*10*) $${\text{CO}}_{\text{max}}=\frac{{\text{SV}}_{\text{max}}}{1,000}\cdot {\text{HR}}_{\text{max}}\;\;(\text{L}\cdot {\text{min}}^{-\text{1}})$$

(*11*) $${\text{CO}}_{\text{rest}}=\frac{{\text{SV}}_{\text{rest}}}{1,000}\cdot {\text{HR}}_{\text{rest}}\;\;\;(\text{L}\cdot {\text{min}}^{-\text{1}})$$

where SV_max_ is the maximum stroke volume (*Eq. 6*); HR_max_ is the maximum heart rate (*Eq. 8*); SV_rest_ is the resting stroke volume at rest (*Eq. 7*); and HR_rest_ is the resting heart rate (*Eq. 9*).

Using these equations, CO_max_ is an indirect function of aerobic capacity, through changes in SV_max_ and age via HR_max_. In support of this approach, a subsample of eight studies (*n* = 25 population groups, total *n* = 251 participants) indicates a strong correlation between SV_max_ (mL·beat^−1^) and CO_max_ (L·min^−1^), with the majority (94%) of the variability in SV_max_ accounted for by changes in CO_max_ (Fig. 1*D*) and vice versa. Of interest, 82% of the variability in CO_max_ was accounted for by changes in V̇o_2max_ (L·min^−1^) (CO_max_ = 4.20 + 5.41·V̇o_2max_) and vice versa. This indicates only 18% of the variability in V̇o_2max_ is accounted for by changes in ${\text{AVO}}_{{\text{2}}_{\text{diffmax}}}$ (14–22).

#### Cardiac output, heart rate, and stroke volume during exercise.

Traditional understanding has been that SV plateaus at exercise intensities above 40%–50% V̇o_2max_ (37). More recently, however, several research studies have demonstrated that SV can, in euhydrated, thermoneutral individuals, increase linearly with exercise intensity up to V̇o_2max_ (17). The proposed progressive increase in SV is thought to be related to enhanced cardiac inotropy, reduced vascular resistance, improved diastolic function, as well as larger blood volumes in endurance-trained athletes, although this has been contested (21, 38).

Importantly, however, the multiplicative function in *Eq. 2* dictates that in situations where a linear increase in SV can be achieved, a nonlinear (i.e., disproportionate) increase in CO up to exhaustion must follow, assuming no plateau in HR would occur. Disproportionate and nonlinear increases in CO with rising exercise intensity would indicate that the heart is distributing increasingly greater proportions of CO to metabolically inactive tissues. This seems unlikely and somewhat counters the notion of linear increases in SV being a beneficial adaptation to elite athletes. Further compounding matter, this phenomenon would also require declines in oxygen extraction above workloads of 60%–70% V̇o_2max_ to maintain linearity between HR and VO_2_. Given this unresolved complexity, the present CVR Model arranged the computation of CO and HR to be linear, with SV therefore forced into a nonlinear relation, as dictated by the CO principle (*Eq. 2*):

(*12*) $$\text{HR}=\left({\text{HR}}_{\text{max}}-{\text{HR}}_{\text{rest}}\right)\cdot {\text{F}\dot{\text{V}}\text{O}}_{2{\text{max}}_{\left(\text{reserve}\right)}}+{\text{HR}}_{\text{rest}}\;(\text{beats}\cdot {\text{min}}^{-\text{1}})$$

(*13*) $$\text{CO}=\left({\text{CO}}_{\text{max}}-{\text{CO}}_{\text{rest}}\right)\cdot {\text{F}\dot{\text{V}}\text{O}}_{2{\text{max}}_{\left(\text{reserve}\right)}}+{\text{CO}}_{\text{rest}}\;(\text{L}\cdot {\text{min}}^{-\text{1}})$$

(*14*) $$\text{SV}=\frac{\text{CO}}{\text{HR}}\cdot 1,000\;(\text{mL}\cdot {\text{beat}}^{-\text{1}})$$

where HR_max_ is the maximum heart rate (*Eq. 8*); HR_rest_ the resting heart rate (*Eq. 9*); CO_max_ the maximum cardiac output value (*Eq. 10*), CO_rest_ the resting cardiac output value (*Eq. 11*); and ${F\dot{V}O}_{2{\text{max}}_{\left(\text{reserve}\right)}}$ the relative fraction of the difference between resting V̇o_2_ and 100% V̇o_2max_ (*Eq. 5*).

### Altitude

As the altitude increases, a reduction in partial pressure of oxygen results in a deoxygenation of arterial blood, and thereby a lower partial pressure of oxygen at the muscle tissue. The net result is a reduced V̇o_2max_, and ultimately a higher relative workload (as a percentage of V̇o_2max_) for any given mechanical work performed at altitude, when compared with the same mechanical work performed at sea level (39–42). Acute systemic hypoxia does not appear to greatly impact the contractile function of cardiac muscle, such that CO_max_ at high altitude is similar to that observed at sea level (43, 44). It can therefore be deduced that the decline in V̇o_2max_ is attributable to reduction in maximum oxygen extraction at the muscle i.e., ${\text{AVO}}_{{\text{2}}_{\text{diffmax}}}$. This is rational, as to maintain absolute oxygen delivery (and thereby oxygen consumption) an increased blood flow is needed to offset a reduced passive oxygen diffusion to the mitochondria. The net result is that for any given oxygen consumption, there is an increased cardiac demand (i.e., CO, HR, and SV) to directly compensate the reduced capacity for oxygen extraction at the muscle (i.e., ${\text{AVO}}_{{\text{2}}_{\text{diff}}}$) (45).

Thus, to effectively model changes in cardiovascular parameters at altitude, it is first necessary to estimate a basic (i.e., not modulated for the environment) maximum oxygen extraction value (${\text{AVO}}_{{\text{2}}_{\text{diffmax}}}$), using the basic CO_max_ (*Eq. 10*) and the V̇o_2max_ of the individual. Resting oxygen extraction (${\text{AVO}}_{{\text{2}}_{\text{diffrest}}}$) and ${\text{AVO}}_{{\text{2}}_{\text{diff}}}$ were expressed correspondingly:

(*15*) $${\text{AVO}}_{2_{\text{diffmax}}}=\;\frac{{\dot{\text{V}}\text{O}}_{2\text{max}}}{{\text{CO}}_{\text{max}}}\;({\text{L}}_{\left[{\text{O}}_{2}\right]}\cdot {{\text{L}}_{\left[\text{blood}\right]}}^{-\text{1}})$$

(*16*) $${\text{AVO}}_{2_{\text{diffrest}}}=\;\frac{{\dot{\text{V}}\text{O}}_{2\text{rest}}}{{\text{CO}}_{\text{rest}}}\;({\text{L}}_{\left[{\text{O}}_{2}\right]}\cdot {{\text{L}}_{\left[\text{blood}\right]}}^{-\text{1}})$$

(*17*) $${\text{AVO}}_{2_{\text{diff}}}=\;\frac{{\dot{\text{V}}\text{O}}_{2}}{\text{CO}}\;\;({\text{L}}_{\left[{\text{O}}_{2}\right]}\cdot {{\text{L}}_{\left[\text{blood}\right]}}^{-\text{1}})$$

where CO_max_ is the basic maximum cardiac output (L·min^−1^; *Eq. 10*); V̇o_2max_ the baseline maximum oxygen consumption of that individual (L·min^−1^); CO_rest_ the basic resting cardiac output (L·min^−1^; *Eq. 11*); and ${\dot{\text{V}}\text{O}}_{2\text{rest}}$ the resting oxygen consumption (L·min^−1^; *Eqs. 4.1* and *4.2*); CO the basic cardiac output (L·min^−1^; *Eq. 13*); and V̇o_2_ the immediate oxygen consumption at that moment of rest or exercise (L·min^−1^).

The degree to which V̇o_2max_ declines for every 1 km gained in altitude has been the focus of meta-analyses, in which Wehrlin and Hallen (40) suggested that V̇o_2max_ declines linearly by 7.7% per km gained in altitude. Fulco et al. (46) on the other hand, suggested a curvilinear decline in V̇o_2max_. In a comprehensive examination of changes in V̇o_2max_ at altitude, a reaggregation of the data of Fulco et al. (46) indicates, when forcing the *Y*-intercept through zero (i.e., no reduction at sea level), that this decline fits the function: decline in V̇o_2max_ = −0.007·km^2^ –0.03·km. On the basis that Fulco et al. (46) utilized: *1*) the widest range of altitudes and *2*) the largest number of observations (*n* = 67), this more complex curvilinear function was used to modulate the CVR Model for altitude. Using the Fulco et al. (46) equation, as well as *Eqs*. *15*–*17* as a basis, *Eqs. 18*–*20* can be used to estimate altitude-induced changes in oxygen extraction. These can in turn be used to modulate CO (*Eq. 21*) for increasing altitudes.

(*18*) $$\begin{array}{l} {\text{AVO}}_{2_{\text{diffmax}\left(\text{altitude}\right)}}=(1-\left(0.007\cdot {\text{km}}^{2}+0.03\cdot \text{km}\right))\cdot {\text{AVO}}_{2_{\text{diffmax}}}({\text{L}}_{\left[{\text{O}}_{2}\right]}\cdot {{\text{L}}_{\left[\text{blood}\right]}}^{-\text{1}}) \end{array}$$

(*19*) $$\begin{array}{l} {\text{AVO}}_{2_{\text{diffrest}\left(\text{altitude}\right)}}=(1-\left(0.007\cdot {\text{km}}^{2}+0.03\cdot \text{km}\right))\cdot {\text{AVO}}_{2_{\text{diffrest}}}({\text{L}}_{\left[{\text{O}}_{2}\right]}\cdot {{\text{L}}_{\left[\text{blood}\right]}}^{-\text{1}}) \end{array}$$

(*20*) $$\begin{array}{l} {\text{AVO}}_{2_{\text{diff}\left(\text{altitude}\right)}}=\frac{\left({\text{AVO}}_{2_{\text{diff}}}-{\text{ AVO}}_{2_{\text{diffrest}}}\right)}{\left({\text{AVO}}_{2_{\text{diffmax}}}-{\text{ AVO}}_{2_{\text{diffrest}}}\right)}\cdot \left({\text{AVO}}_{2_{\text{diffmax}\left(\text{altitude}\right)}}-{\text{AVO}}_{2_{\text{diffrest}\left(\text{altitude}\right)}}\right)+{\text{AVO}}_{2_{\text{diffrest}\left(\text{altitude}\right)}}({\text{L}}_{\left[{\text{O}}_{2}\right]}\cdot {{\text{L}}_{\left[\text{blood}\right]}}^{-\text{1}}) \end{array}$$

(*21*) $${\text{CO}}_{\left(\text{altitude}\right)}=\frac{{\dot{\text{V}}\text{O}}_{2}}{{\text{AVO}}_{2_{\text{diff}\left(\text{altitude}\right)}}}\;(\text{L}\cdot {\text{min}}^{-\text{1}})$$

where ${\text{AVO}}_{{\text{2}}_{\text{diffmax}}}$ is the maximum arterial oxygen extraction at sea level (*Eq. 15*); ${\text{AVO}}_{2_{\text{diffrest}}}$ the resting arterial oxygen extraction at sea level (*Eq. 16*); ${\text{AVO}}_{{\text{2}}_{\text{diff}}}$ the arterial oxygen extraction at sea level (*Eq. 17*); and “km” the altitude above sea level in kilometers.

### Heat and Dehydration

#### Stroke volume during passive heating at rest and during exercise-induced heat strain.

In the heat, cardiac filling pressure is reduced, largely due to the lower peripheral vascular resistance with increases in skin blood flow necessary for thermoregulation. The net result is a lowering of SV, necessitating a compensatory increase in HR, to maintain the CO required for both exercise and thermoregulation (47). Thus, to modulate cardiac strain with heat stress, it is necessary to assess the effect of heat on SV during both rest and exercise across a range of exercise intensities.

**Figure 2.**
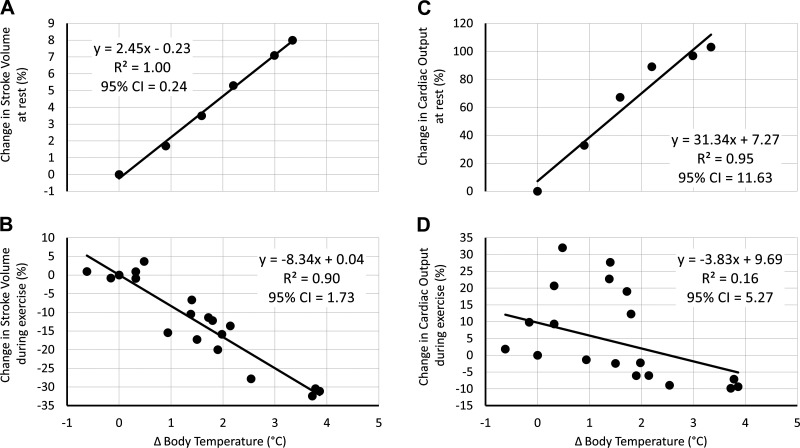
*A*: increase in stroke volume (%) during resting passive heat stress. *B*: decline in stroke volume (%) during exercising heat stress. *C*: increase in cardiac output (%) during resting passive heat stress. *D*: decline in cardiac output (%) during exercising heat stress. Mean body temperature (°C) is calculated as follows: 0.2 × *T*_skin_ + 0.8 × *T*_core_. Data in *A* and *C* are taken and reanalyzed from Rowell (47) (*n* = 12). Data in *B* and *D* are taken and reanalyzed from Rowell et al. (52, 53) and González-Alonso et al. (54) (*n* = 28).

In a meta-analysis of data from several publications using direct passive heating to resting individuals (48–51), reaggregation and analysis of Rowell (41) demonstrated a strong linear relation (*R*^2^ > 0.99; Fig. 2*A*) between the increase in SV, and the increase in the mean body temperature (*T*_body_; calculated as *T*_body_ = 0.2 + *T*_skin_ + 0.8 + *T*_core_). Of note, both mean skin (*T*_skin_) and core temperature (*T*_core_) increased nonlinearly, indicating neither variable on its own is the key causative factor behind the increase in SV during passive heating at rest [see also: Fig. 4 in Rowell (47)]. The results indicated that SV increases by 2.45%·°C^−1^ increase in *T*_body_ at rest. Similarly, across the range of mild to exhaustive exercise intensities, reaggregation and analysis of studies by Rowell et al. (52, 53) and González-Alonso et al. (54), indicate that exercise SV declines linearly with increasing *T*_body_ at a rate of 8.3%·°C^−1^ increase in *T*_body_; a decline that is largely independent of the exercise intensity (Fig. 2*B*). Much like passive heating, the relative changes in SV during exercise in the heat appear to be best described by *T*_body_ (*R*^2^ = 0.90), as opposed to *T*_core_ (*R*^2^ = 0.78) or *T*_skin_ (*R*^2^ = 0.40) per se.

#### Cardiac output during heat strain at rest and during exercise.

As well as modulations in SV, it is known that CO will also independently increase with heat stress during both rest and during exercise (47). Thus, by repeating the procedures noted above for SV, CO responses to passive heating at rest, and during exercise, were examined (48–54). The results indicated that at rest, heat stress resulted in a 31% increase in CO·°C^−1^ increase in *T*_body_ (*R*^2^ of 0.95; Fig. 2*C*). In relative terms, the increase in CO is substantially larger than the increases observed in SV; hence a compensatory increase in HR (i.e., cardiovascular drift) is required to match the increased demand for CO during passive heating (47).

**Figure 3.**
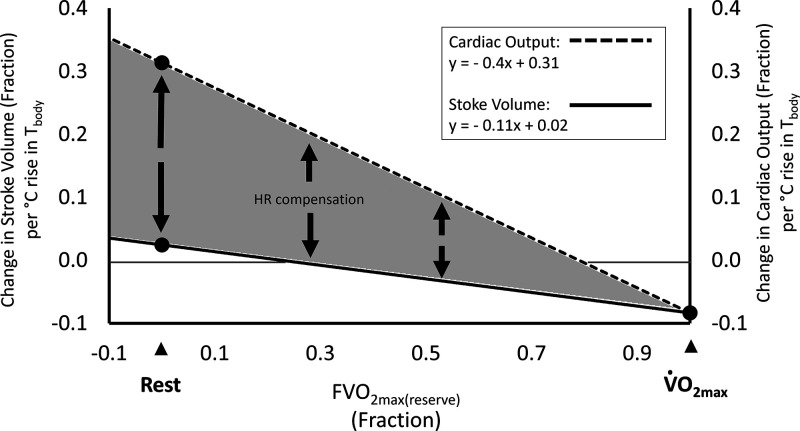
Modulations in stroke volume and cardiac output for every 1°C increase in *T*_body_, where zero on the primary and secondary *y*-axes represents the basic cardiovascular response (CVR) model cardiac output (CO) and stroke volume (SV) values, respectively, as defined in *Eqs. 13* and *14*. Data taken and reanalyzed from Rowell et al. (48–51), Detry et al. (48), and González-Alonso et al. (54). The gray area represents the discrepancy between cardiac output and stroke volume changes in the heat that must be compensated for by an increase in heart rate.

Although no clear relation was observed in the change in CO with changing body temperatures, across low to high exercise intensities (Fig. 2*D*), the aggregated exercise data indicated that CO either remains slightly elevated, or marginally decreased (range: 15% to −3%) compared with control, during submaximal exercise in the heat (52–54). This is dissimilar to SV where clear declines are consistently observed. At the point of exhaustion, a clear reduction in CO was observed, likely due to a reduction in SV_max_ with little or no change in HR_max_ (Fig. 3).

#### Heat sensitivity of cardiac out and stroke volume.

In summary, the aforementioned observations (48–54) indicate that SV_rest_ increases by 2.5%·°C^−1^ increase in *T*_body_, whereas CO_rest_ increases by 31.3%·°C^−1^ increase in *T*_body_. Both SV_max_ and CO_max_ decrease by −8.3%·°C^−1^ at the point of V̇o_2max_. Using these values, Fig. 3 demonstrates the impacts of a 1°C increase in *T*_body_ above thermoneutral values on both SV and CO, across the full range of aerobic exercise intensities.

#### Dehydration effects on stroke volume and cardiac output.

A limitation of the aforementioned sensitivities is that they represent change in *T*_body_, but do not consider the increase in dehydration that typically accompanies hyperthermia (55–57). In a carefully designed study, González-Alonso et al. (57) demonstrated that in well-trained runners, hydration level operates interactively with hyperthermia to induce reductions in SV during exercise in the heat. In their study, the authors reported a decline in SV·1°C^−1^ increase in *T*_body_ of 0.6%·1°C^−1^ when euhydrated, but 7.2%·1°C^−1^ when severely dehydrated [Fig. 6 in González-Alonso et al. (57)]. These findings indicate the interaction between body temperature and hydration level on cardiac function is, at least in part, synergistic (41, 55, 58). It is important to recognize, however, that the work of González-Alonso et al. (57) was conducted on young endurance-trained male runners (V̇o_2max_ = 3.6 ± 1.1 L·min^−1^). It is possible that individuals less adapted to physical exercise would exhibit a greater sensitivity to increases in *T*_body_, i.e., hyperthermia independently of dehydration per se. Thus, a simplified method of integrating dehydration percentage into the cardiac heat sensitivities, in which Δ*T*_body_ and dehydration contribute additively, was also explored (41, 58).

#### Calculating the synergistic modulations of cardiac heat strain.

To understand the relative contributions of changes in *1*) *T*_body_; *2*) dehydration; and *3*) the synergistic interaction between these factors (i.e., Δ*T*_body_ × dehydration) to the decline in SV, multiple linear regression without a constant was conducted on the SV data presented by González-Alonso et al. (57) (SPSS v. 24.0, IBM). Relative contributions were expressed as the explained variance (adjusted *r*^2^ = 0.99), distributed over each parameter according to their standardized regression coefficients (Δ*T*_body_ = 0.095; percentage dehydration = 0.591; interaction = 0.386), relative to the sum of the standardized regression coefficients (sum = 1.072) (10). The results indicated that changes in *T*_body_ accounted for 8.8%, dehydration percentage 54.9%, and the synergistic interaction 35.8% of the total 28 mL decline in SV reported by González-Alonso et al. (57).

To scale heat-induced changes in SV and CO according to the relative contributions of dehydration, hyperthermia, and the synergism between these factors, a cardiac heat strain index (CHSI) was proposed. This CHSI was organized to directly replace Δ*T*_body_ when using the heat sensitivities defined in *Eqs. 23–26* thus, it was necessary to first normalize the decline in SV reported by González-Alonso et al. (57) to range between 0 (no change in SV) and 1 (maximum decline in SV), then multiply these values by the maximum change in Δ*T*_body_ used to develop *Eqs. 23–26* [i.e., +3.9°C; Fig. 2*B*; González-Alonso et al. (54)]. This adjustment ensures that CHSI is appropriately scaled to the range and changes in Δ*T*_body_ used in the model development. Using the unstandardized regression coefficients, without a constant, the following synergistic CHSI was developed (*Eq. 22*, alternative 1).

(*22.1*) $${\text{CHSI}}_{\left(\text{synergistic}\right)}=0.433\cdot {\text{\%}}_{\text{Dehyd}}+0.091\cdot \Delta T_{\text{body}}+0.125\cdot ({\text{\%}}_{\text{Dehyd}}\cdot \Delta T_{\text{body}})\;\;\;\left(\text{Fraction}\right)$$

where Δ*T*_body_ is the increase in mean body temperature (°C) above a reference of point of 36.54°C, calculated as 0.2·*T*_skin_ + 0.8·*T*_core_ – 36.54 (4); and “%_Dehyd_” is the dehydration percentage, calculated as: (weight loss/weight) × 100. Note: A CHSI value of 3.9 provides the equivalent change in stroke volume (and cardiac output) to that of 3.9°C increase in mean body temperature when using the heat sensitivities defined in *Eqs. 23–26.*

#### Calculating the additive modulations of cardiac heat strain.

To develop the alternative additive CHSI model, in which cardiac strain exhibits a sensitivity to increases in *T*_body_ independently of dehydration per se, a simple equal contribution from Δ*T*_body_ and dehydration was used (*Eq. 22*, alternative 2). Both the additive and synergistic CHSI calculations were used in the first validation data set to compare for accuracy.

(*22.2*) $${\text{CHSI}}_{\left(\text{additive}\right)}={\%}_{\text{Dehyd}}\cdot 0.5+\Delta T_{\text{body}}\cdot 0.5\;\left(\text{Fraction}\right)$$

where Δ*T*_body_ is the increase in mean body temperature (°C) above a reference of point of 36.54°C, calculated as 0.2·*T*_skin_ + 0.8·*T*_core_ – 36.54 (4); and “%_Dehyd_” is the dehydration percentage, calculated as: (weight loss/weight) × 100.

#### Model development to predict stroke volume and cardiac output during heat strain.

Using the heat sensitivities summarized in Fig. 3, and either of the alternative CHSI equations aforementioned, the following equations can be produced for heat-induced modulations of the basic SV_max_ (*Eq. 6*), CO_max_ (*Eq. 10*), SV_rest_ (*Eq. 7*), and CO_rest_ (*Eq. 11*) values:

(*23*) $${\text{SV}}_{{\text{max}}_{\left(\text{heat},\text{altitude}\right)}}=(1+\text{CHSI}\times -0.083)\cdot {\text{SV}}_{\text{max}}\;(\text{mL}\cdot {\text{beat}}^{-\text{1}})$$

(*24*) $${\text{CO}}_{{\text{max}}_{\left(\text{heat},\text{altitude}\right)}}=(1+\text{CHSI}\times -0.083)\cdot {\text{CO}}_{\text{max}}\;(\text{L}\cdot {\text{min}}^{-\text{1}})$$

(*25*) $${\text{SV}}_{{\text{rest}}_{\left(\text{heat},\text{alitude}\right)}}=(1+\text{CHSI}\times 0.025)\cdot {\text{SV}}_{\text{rest}}\;(\text{mL}\cdot {\text{beat}}^{-\text{1}})$$

(*26*) $${\text{CO}}_{{\text{rest}}_{\left(\text{heat},\text{altitude}\right)}}=(1+\text{CHSI}\times 0.313)\cdot {\text{CO}}_{\text{rest}}(\text{L}\cdot {\text{min}}^{-\text{1}})$$

where CHSI is the cardiac heat strain index (*Eq. 22*); −0.083 represents the relative change in maximum cardiac output and maximum stroke volume with every 1°C increase in *T*_body_ and associated dehydration. SV_max_ is the CVR Model’s basic maximum stroke volume value (*Eq. 6*); CO_max_ the CVR Model’s basic maximum cardiac output value (*Eq. 10*); 0.025 the relative change in resting stroke volume with every 1°C increase in *T*_body_ and associated dehydration; 0.313 the relative change in resting cardiac output with every 1°C increase in *T*_body_ and associated dehydration; SV_rest_ is the CVR Model’s basic resting stroke volume value (*Eq. 7*); and CO_rest_ the CVR Model’s basic resting cardiac output value (*Eq. 11*).

In these equations, ${\text{CO}}_{{\text{max}}_{\left(\text{heat},\text{altitude}\right)}}$ is the heat, dehydration, and altitude-modulated value for the CVR Model’s basic CO_max_ (*Eq. 10*), whereas ${\text{SV}}_{{\text{max}}_{\left(\text{heat},\text{altitude}\right)}}$ is the heat, dehydration, and altitude-modulated value of the CVR Model’s basic SV_max_ (*Eq. 6*). Similarly, ${\text{CO}}_{{\text{rest}}_{\left(\text{heat},\text{altitude}\right)}}$ is the heat, dehydration, and altitude-modulated value for the CVR Model’s basic CO_rest_ (*Eq. 11*), and ${\text{SV}}_{{\text{rest}}_{\left(\text{heat},\text{alitude}\right)}}$ is the heat, dehydration, and altitude-modulated value of the CVR Model’s basic SV_rest_ (*Eq. 7*). Of note, *Eqs. 23–26* dictate that during passive (rested) heating, both SV_rest_ and HR_rest_ must increase to compensate the higher requirement for CO_rest_. In contrast, due to the equal sensitivity of CO_max_ and SV_max_ to heat, these equations mandate that the decline in CO_max_ is entirely accounted for by changes in SV_max_ (Fig. 3), with no change in HR_max_. Together, these equations reflect a combined inotropic and chronotropic adaptation to heat at rest, and a strictly inotropic limitation imposed by heat at V̇o_2max_. A new HR_rest_ value for heat is therefore necessitated, whereas an updated HR_max_ value is not required. HR resting in the heat can therefore be calculated as follows:

(*27*) $${\text{HR}}_{{\text{rest}}_{\left(\text{heat},\text{altitude}\right)}}=\frac{{\text{CO}}_{{\text{rest}}_{\left(\text{heat},\text{altitude}\right)}}}{{\text{SV}}_{{\text{rest}}_{\left(\text{heat},\text{altitude}\right)}}}(\text{beat}\cdot {\text{min}}^{-\text{1}})$$

where ${\text{SV}}_{{\text{rest}}_{\left(\text{heat},\text{altitude}\right)}}$ is the resting stroke volume in the heat (*Eq. 25*); and ${\text{CO}}_{{\text{rest}}_{\left(\text{heat},\text{altitude}\right)}}$ the resting cardiac output in the heat (*Eq. 26*).

#### Changes to relative workloads in the heat and at altitude.

The respective changes in CO and CO_max_ caused by altitude and heat require an update in the relative workload calculation for the CVR Model. Thus, the workload (defined as fraction of the difference between V̇o_2rest_ and V̇o_2max_) is recalculated in *Eq. 28*, accounting for the cardiac modulations caused by both altitude and heat stress.

(*28*) $${\text{F}\dot{\text{V}}\text{O}}_{2{\text{max}}_{\left(\text{reserve},\text{heat},\text{altitude}\right)}}=\frac{{\text{ CO}}_{\left(\text{altitude}\right)}-{\text{CO}}_{\text{rest}}}{{\text{CO}}_{{\text{max}}_{\left(\text{heat},\text{altitude}\right)}}-{\text{CO}}_{\text{rest }}}\left(\text{Fraction}\right)$$

where CO_rest_ is the basic model resting cardiac output value (*Eq. 11*); CO_(altitude)_ the cardiac output when modulated for altitude only (*Eq. 21*); and ${\text{CO}}_{{\text{max}}_{\left(\text{heat},\text{altitude}\right)}}$ the maximum cardiac output modulated for heat, dehydration, and altitude (*Eq. 24*).

Using the values for ${\text{F}\dot{\text{V}}\text{O}}_{2{\text{max}}_{\left(\text{reserve},\text{heat},\text{altitude}\right)}}$, ${\text{SV}}_{{\text{max}}_{\left(\text{heat},\text{altitude}\right)}}$, ${\text{CO}}_{{\text{max}}_{\left(\text{heat},\text{altitude}\right)}}$, ${\text{SV}}_{{\text{rest}}_{\left(\text{heat},\text{altitude}\right)}}$, ${\text{CO}}_{{\text{rest}}_{\left(\text{heat},\text{altitude}\right)}}$, and ${\text{HR}}_{{\text{rest}}_{\left(\text{heat},\text{altitude}\right)}}$ (*Eqs. 23–28*), the heat, dehydration, and altitude modulated SV, CO, and HR are then calculated as:

(*29*) $$\begin{array}{l} {\text{HR}}_{\left(\text{heat},\text{altitude}\right)}=\left({\text{HR}}_{\text{max}}-{\text{HR}}_{{\text{rest}}_{\left(\text{heat},\text{altitude}\right)}}\right)\cdot \text{F}\dot{\text{V}}{\text{O}}_{{2\text{max}}_{\left(\text{reserve},\text{heat},\text{altitude}\right)}}+{\text{HR}}_{{\text{rest}}_{\left(\text{heat},\text{altitude}\right)}}\\ \left(\text{beats}\cdot {\text{min}}^{-\text{1}}\right) \end{array}$$

(*30*) $$\begin{array}{l} {\text{CO}}_{\left(\text{heat},\text{altitude}\right)}=\left({\text{CO}}_{{\text{max}}_{\left(\text{heat},\text{altitude}\right)}}-{\text{CO}}_{{\text{rest}}_{\left(\text{heat},\text{altitude}\right)}}\right)\cdot \text{F}\dot{\text{V}}{\text{O}}_{2{\text{max}}_{\left(\text{reserve},\text{heat},\text{altitude}\right)}}+{\text{CO}}_{{\text{rest}}_{\left(\text{heat},\text{altitude}\right)}}(\text{L}\cdot {\text{min}}^{-\text{1}}) \end{array}$$

(*31*) $${\text{SV}}_{(\text{heat},\text{altitude})\;}=\;\frac{{\text{CO}}_{\left(\text{heat},\text{altitude}\right)}}{{\text{HR}}_{\left(\text{heat},\text{altitude}\right)}}\cdot \;1,000\;(\text{mL}\cdot {\text{beat}}^{-\text{1}})$$

where HR_max_ is the maximum heart rate (*Eq. 8*); ${\text{HR}}_{{\text{rest}}_{\left(\text{heat},\text{altitude}\right)}}$ the heat, dehydration, and altitude modulated value for the CVR Model’s basic resting heart rate (*Eq. 27*); ${F\dot{V}O}_{2{\text{max}}_{\left(\text{reserve},\text{heat},\text{altitude}\right)}}$ relative fraction of the difference between resting V̇o_2_ and 100% V̇o_2max_ (*Eq. 28*); and ${\text{CO}}_{{\text{max}}_{\left(\text{heat},\text{altitude}\right)}\;}$ the heat, dehydration, and altitude modulated for the CVR Model’s basic CO_max_ (*Eq. 24*); ${\text{CO}}_{{\text{rest}}_{\left(\text{heat},\text{altitude}\right)}}$ is the heat, dehydration, and altitude modulated value for the CVR Model’s basic resting cardiac output (*Eq. 26*).

In these equations, HR_(heat,altitude)_ is the heat, dehydration, and altitude modulated value of the CVR Model’s basic HR (*Eq. 12*); CO_(heat,altitude)_ reflects the heat, dehydration, and altitude modulated value for the CVR Model’s basic CO (*Eq. 13*); and SV_(heat,altitude)_ is the heat, dehydration, and altitude modulated of the CVR Model’s basic SV (*Eq. 14*).

### Impact of Heat and Altitude on Aerobic Capacity

The reduction in CO_max_ during acute exposure to heat and dehydration, as well as the reduction in ${\text{AVO}}_{{\text{2}}_{\text{diffmax}}}$ during acute exposure to altitude, are not compensated by an increase in HR_max_ (43, 54). Thus, the net outcome must be a reduction in V̇o_2max_, according to the Fick principle. This is demonstrated empirically by studies examining the effects of altitude and progressive heat-strain on V̇o_2max_ (59, 60). Using *Eq. 32*, a V̇o_2max_ value that reflects the environmental strains imposed by heat and altitude (i.e., ${\dot{\text{V}}\text{O}}_{2{\text{max}}_{\left(\text{heat},\text{altitude}\right)}}$), can be calculated as:

(*32*) $${\dot{\text{V}}\text{O}}_{2{\text{max}}_{\left(\text{heat},\text{altitude}\right)}\;}={\text{AVO}}_{2{\text{diffmax}}_{\left(\text{altitude}\right)}}\cdot {\text{CO}}_{{\text{max}}_{\left(\text{heat},\text{altitude}\right)}}\;(\text{L}\cdot {\text{min}}^{-\text{1}})$$

where ${\text{AVO}}_{2{\text{diffmax}}_{\left(\text{altitude}\right)}}$ is the maximum arterial oxygen extraction at altitude (*Eq. 18*); and ${\text{CO}}_{{\text{max}}_{\left(\text{heat},\text{altitude}\right)}}$ the heat, dehydration, and altitude-modulated value for the CVR Model’s basic CO_max_ (*Eq. 24*).

It is important to note that the effect of heat and dehydration on V̇o_2max_ (via changes in SV_max_ and CO_max_) in the present CVR model is based on equations derived from a series of basic physiological studies examining cardiac function. Thus, it is useful to compare the outcomes of this prediction to other empirical data examining measured changes in V̇o_2max_ during exercise in the heat. Aggregation of study data by Périard and Racinais (60) indicates a linear decline in V̇o_2max_ of 11.9%·C^−1^ increase in *T*_body_ (*R*^2^ = 0.91). Using the Havenith et al. (11) validation data set detailed below (in *Validation 1: Heart rate responses to rest and exercise in the heat*), the CVR Model with the additive CHSI predicted an almost identical decline in V̇o_2max_ to the 11.9%·°C^−1^ observed by Périard and Racinais (60) (*y* = 0.97*x* + 0.18; *R*^2^ = 0.98).

### Redistribution of Cardiac Output

The CVR Model provides a refined estimate of CO with increasing exercise intensity, altitude, body temperature, and dehydration. The model can therefore also be used to estimate the proportion of CO required for regional blood perfusion rates in skin tissue, active muscles, and visceral organs during exercise, across different environmental conditions. To achieve this, the required core blood flow (CoreBF) at rest and during exercise was first estimated. While Stolwijk (6) estimated CoreBF to be 4.5 L·min^−1^ for major organs at rest, Williams and Leggett (61) estimated a value of 70% of CO_rest_. Rowell (47), however, provided a dynamic (inverse exercise intensity dependent) estimate of CoreBF (e.g., Fig. 8 in Ref. 47). Following reaggregation of the data by Rowell (47), CoreBF (as fraction of total CO) appeared to vary nonlinearly and inversely correlated (CoreBF = 0.86·(CO/CO_max_)^2^ – 1.79·(CO/CO_max_) + 1.06, *R*^2^ = 0.99) with the relative demand placed on the heart (i.e., CO as a fraction of true CO_max_), irrespective of the environmental condition in which exercise was performed (47). Thus, the following equation was used to estimate required CoreBF.

(*33*) $$\begin{array}{l} \text{CoreBF}={\text{CO}}_{\left(\text{heat},\text{altitude}\right)}\cdot (0.86\cdot {{\text{FCO}}_{{\text{max}}_{\left(\text{heat},\text{altitude}\right)}}}^{2}-1.79\cdot \;{\text{FCO}}_{{\text{max}}_{\left(\text{heat},\text{altitude}\right)}}+1.06)(\text{L}\cdot {\text{min}}^{-\text{1}}) \end{array}$$

where CO_(heat,altitude)_ is the cardiac output modulated for heat, dehydration, and altitude (L·min^−1^; *Eq. 30*); and FCO_max_ the relative demand placed on the heart (i.e., CO_(heat,altitude)_/${\text{CO}}_{{\text{max}}_{\left(\text{heat},\text{altitude}\right)}}$).

Skin blood flow (SkBF) was estimated using an assumed resting, thermoneutral skin blood flow of 5% of cardiac output (61), plus the excess blood flow caused by increases in body temperature and dehydration (i.e., the difference between CO_(altitude)_ and CO_(heat,altitude)_). It should be recognized that when coupling thermoregulatory models with the CVR model, precise SkBF estimation is paramount to modeling heat transfer within the body, and between the body and the external environment. Thus, the calculation of SkBF (*Eq. 34*) may be better implemented as a constraint on SkBF necessary for thermoregulation, rather than a direct value substitution per se, thereby limiting the CVR Models’ interference with the intricacies of the heat exchange estimations.

(*34*) $$\text{SkBF}=(0.05\times {\text{CO}}_{\text{rest}})+\left({\text{CO}}_{\left(\text{heat},\text{altitude}\right)}-{\text{CO}}_{\left(\text{altitude}\right)}\right)\;(\text{L}\cdot {\text{min}}^{-\text{1}})$$

where CO_rest_ is the CVR Model’s basic resting cardiac output value (*Eq. 11*); CO_(heat,altitude)_ the cardiac output modulated for heat, dehydration, and altitude (L·min^−1^; *Eq. 30*); and CO_(altitude)_ the cardiac output when modulated for altitude only (*Eq. 21*).

Required muscle blood flow (MusBF) was also calculated by subtracting SkBF (*Eq. 34*) and CoreBF (*Eq. 33*) from CO_(heat,altitude)_ (*Eq. 30*).

(*35*) $$\text{MusBF}={\text{CO}}_{\left(\text{heat,altitude}\right)}-\text{SkBF}-\text{CoreBF }(\text{L}\cdot {\text{min}}^{-\text{1}})$$

where CO_(heat,altitude)_ is the cardiac output modulated for heat, dehydration, and altitude (L·min^−1^; *Eq. 30*); “CoreBF” the core blood flow (L·min^−1^; *Eq. 33*); and “SkBF” the skin blood flow (L·min^−1^; *Eq. 34*).

Importantly, MusBF can also be used to provide a variable (as opposed to a fixed) active muscle oxygen extraction (i.e., Muscle ${\text{AVO}}_{{\text{2}}_{\text{diff}}}$) across exercise intensities, systemic oxygen concentration levels, and thermophysiological states. This approach contrasts the use of a predefined oxygen extraction value of 200 m${\text{L}}_{\left[{\text{O}}_{2}\right]}$·L_[blood]_^−1^, which is then used to estimate CO in several complex thermophysiological models.

(*36*) $${\text{Active Muscle AVO}}_{2_{\text{diff}}}=\frac{\left({\dot{\text{V}}\text{O}}_{2}-{\dot{\text{V}}\text{O}}_{2\text{rest}}\right)}{\text{MusBF}}({\text{L}}_{\left[{\text{O}}_{2}\right]}\cdot {{\text{L}}_{\left[\text{blood}\right]}}^{-\text{1}})$$

where V̇o_2_ is the time-variable oxygen consumption during rest or exercise (L·min^−1^); ${\dot{V}O}_{2\text{rest }}$ the individual’s resting oxygen consumption (*Eqs. 4.1* and *4.2*; L·min^−1^); and MusBF the active muscle blood flow (*Eq. 35*).

### Exercise Limitations and Task Failure

An additional utility of the present model is the capacity to estimate the sustainability of a physical task. When the exercising workload necessitates either V̇o_2_, HR_(heat,altitude)_, CO_(heat,altitude)_, *T*_body_, dehydration that is greater than the maximum value achievable or tolerable, the model reports that the exercise is no longer aerobically sustainable, and task failure occurs. Lower HR_max_ thresholds and/or body temperature thresholds can also be applied to simulate, e.g., occupational work tolerability.

## RESULTS

The key inputs used for the validation of the CVR Model were personal characteristics of the simulated individual(s), including baseline V̇o_2max_, age, body mass, height, and sex as well as information on the actual exposure including altitude, exercise time, workload V̇o_2_, total exercise time, the total body mass loss, and *T*_body_. For all validation purposes, progressive dehydration level was approximated by linear interpolation using *Eq. 37* and used in the calculation of the CHSI.

(*37*) $$\text{\%Dehydration}=\frac{\text{Exercise Time Elapsed}}{\text{Total Exercise Time}}\times \text{Total Mass Loss}\;\;\;\;\left(\%\right)$$

where total mass loss is the total body mass lost at the end of the exercise period (kg); and exercise time measured in minutes.

### Graded Exercise Test Simulations

#### A simulated incremental exercise test to exhaustion.

**Figure 4.**
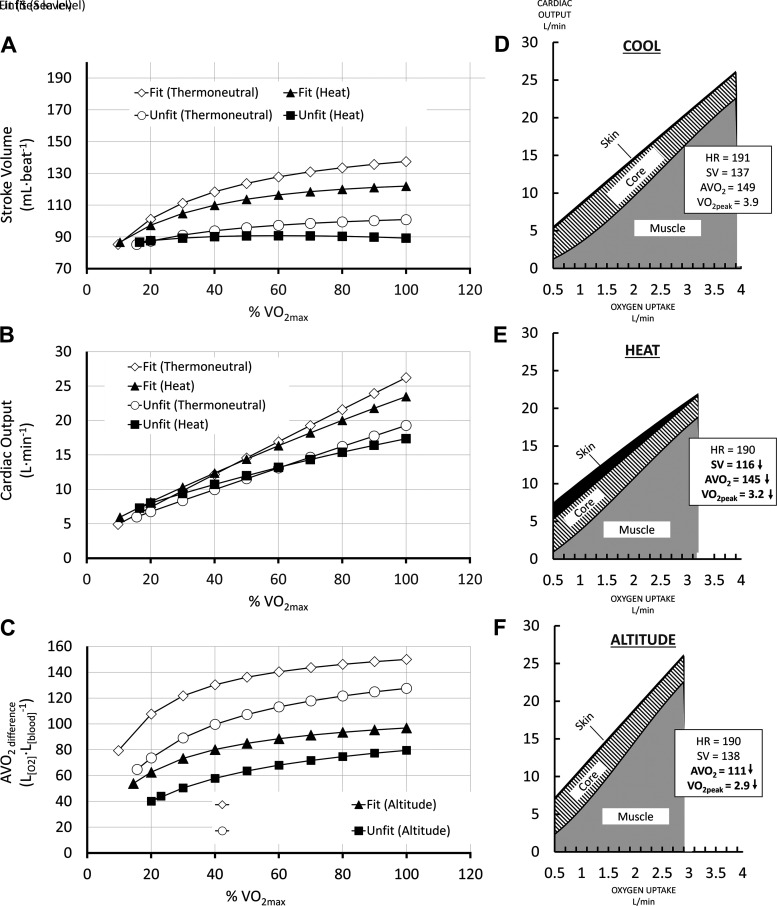
*A* and *B*: cardiac output and stroke volume of a fit (V̇o_2max_ = 4.0 L·min^−1^) and unfit (V̇o_2max_ = 2.5 L·min^−1^) during a simulated graded exercise test to exhaustion in thermoneutral and heated conditions. *C*: oxygen extraction response of a fit (V̇o_2max_ = 4.0 L·min^−1^) and unfit (V̇o_2max_ = 2.5 L·min^−1^) individual during a simulated graded exercise test to exhaustion in sea level and high-altitude conditions. *D*–*F*: fit (V̇o_2max_ = 4.0 L·min^−1^) individuals compartmental blood flows with increasing exercise intensity under thermoneutral, heated, and high-altitude conditions. Simulated individuals were 25 yr, 75 kg, and 175 cm. Simulated tests were at rest, followed by exercise at 20% to 100% V̇o_2max_ in steps of 10%/2-min exercise time. Thermoneutral conditions were 36.6°C *T*_body_ and 0.25 kg mass loss. Heated conditions were 39°C *T*_body_ and 1.5 kg mass loss. Sea level and high-altitude conditions were 0-km vs. 5-km altitude, respectively.

Figure 4 provides the results of a simulated exercise test, in which a fit and an unfit individual (with all other characteristics equal) completed rest, followed by exercise at 20 through to 100% V̇o_2max_. The results show the change in SV and CO (Fig. 4, *A* and *B*) in a thermoneutral and heated conditions. The changes in ${\text{AVO}}_{{\text{2}}_{\text{diff}}}$ are also provided (Fig. 4*C*) for sea level and high-altitude conditions. Compartmental blood flow is also illustrated for the fit individual at sea level, heated, and high-altitude conditions (Fig. 4, *D*–*F*).

### Heart Rate Validation

Predicted HR in the heat, i.e., HR_(heat,altitude)_ (*Eq. 29*) provides a quantitative parameter on which to validate cardiovascular strain, whereby predicted HR can be compared with a valid, reliably measured, and widely reported parameter in healthy populations. Predicted HR is therefore particularly useful for validation purposes, as inaccuracies in the underlying SV or CO values would result in erroneous predicted HR values also.

#### Validation 1: Heart rate responses to rest and exercise in the heat.

A validation of the CVR Model’s HR prediction (i.e., cardiac strain) was conducted using a data set of heterogeneous individuals during rest and exercise in the heat (10, 11). Two validation data sets were combined to conduct this validation. The first validation data set examined cardiovascular and thermoregulatory responses in a population of 45 individuals, varied by age, aerobic fitness level, and body morphology (age: 30–73 yr; V̇o_2max_ 1.8–4.4 L·min^−1^; mass: 49.8–104.6 kg; body fat: 8.2%–40.4%; height: 157.1–189.7 cm), during low-intensity cycling at a fixed intensity of 60 W, for 1 h, in a warm-humid environment (35°C, 80% relative humidity) (11). The data include 30-min rest in the experimental climate, followed by 60-min exercise, with the measured data reported every 5 min (total number of data points = 807). The second validation data set examined cardiovascular and thermoregulatory responses during two different exercise modalities, and three different climates (10). The first exercise modality was conducted by 26 individuals (age: 18–35 yr; V̇o_2max_ 1.9–5.3 L·min^−1^; mass: 49.8–102.1 kg; body fat: 7.3%–32.3%; height: 162.0–193.5 cm) using low-intensity cycling at a fixed power output of 60 W, for 1 h, in a warm-humid environment (35°C, 80% relative humidity). The second exercise modality was conducted by a population of 24 young individuals (age: 20–27 yr; V̇o_2max_ 2.0–4.1 L·min^−1^; mass: 63.3–85.9 kg; body fat: 12.6%–23.0%; height: 177–193 cm) at rest, followed by cycling at 25%, then 45% V̇o_2max_, in three different climates (Cool: 21°C, 50% relative humidity; Warm-Humid: 35°C, 80% relative humidity; and Hot-Dry: 45°C, 20% relative humidity). These data also include 30-min rest in the experimental climate, followed by 60-min exercise, with data reported every 30 min (total number of data points = 294). Both the synergistic and additive (*Eq. 22*) CHSI were compared for predictive power using this data set.

**Figure 5.**
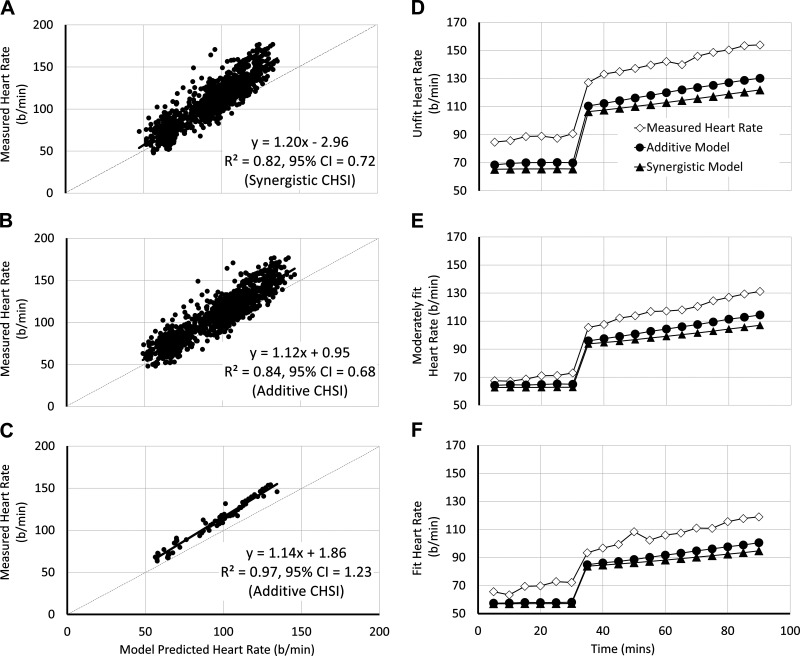
*A* and *B*: relationship between the cardiovascular response (CVR) model heart rate prediction (*Eq. 29*), when using the synergistic and additive cardiac heat strain index (CHSI), respectively (*Eq. 22*), against measured heart rate data, on 96 individuals, during rest, and exercise in the heat. *C*: relationship between the CVR model heart rate prediction (*Eq. 29*) using the additive CHSI (*Eq. 22*) against the measured heart rate data stratified into groups means based on aerobic fitness level and climate type. Fitness levels were stratified as: unfit = <3 L·min^−1^ V̇o_2max_; moderate = 3–4 L·min^−1^ V̇o_2max_; and fit = >4 L·min^−1^ V̇o_2max_. Climate types were stratified as follows: cool = 21°C, 50% relative humidity; warm-humid = 35°C, 80% relative humidity; and hot-dry = 45°C, 20% relative humidity. *D*–*F*: compares the change in predicted and measured heart rate over time in unfit, moderately fit, and fit individuals, when using the synergistic and additive CHSI (*Eq. 22*). Measured data are taken and reanalyzed from Havenith (11) (*n* = 45) and Havenith (10) (*n* = 50).

Measured heart rate (10, 11) was then compared with predicted HR (*Eq*. *29*). The results of the validation indicate a strong relationship (Fig. 5, *A* and *B*; *R*^2^ = 0.82–0.84) between measured and predicted HR, on the individual data and when using both the additive and synergistic models. When the data were stratified into group means by aerobic fitness level (Unfit = <3 L·min^−1^ V̇o_2max_; Moderate = 3–4 L·min^−1^ V̇o_2max_; Fit = >4 L·min^−1^ V̇o_2max_) and by climate (relative-intensity exercise in Cool, Warm-Humid, Hot-Dry, and fixed-intensity exercise in Warm-Humid), the agreement between measured and predicted HR is increased further (Fig. 5*C*; *R*^2^ = 0.97), but was underestimated across all fitness levels and climates (Fig. 5, *D*–*F*). Although both the synergistic and additive (*Eq. 22*) CHSI provided strong predictions, the additive model provided an improved prediction across all fitness levels (Fig. 5, *D*–*F*). This contrasts the expectation that the synergistic CHSI would better predict fitter individuals HR response since the synergistic CHSI was produced using a data set of endurance-trained runners (57).

#### Validation 2: Heart rate responses to rest and exercise at altitude.

A validation of the CVR Model’s HR prediction was also conducted using a smaller data set of individuals at rest and during exercise at altitude (Tsuji et al., 2019, unpublished observations). The data set examined cardiovascular and thermoregulatory responses in six males (age: 21–32 yr; V̇o_2max_ 2.9–3.8 L·min^−1^; mass: 59.0–83.5 kg; height: 166–186 cm) during rest and exercise in a cool high-altitude environment (20°C, 40% relative humidity, 12.7% inspired O_2_). The data included 30-min rest in the experimental climate, followed by 30-min low-intensity cycling at 35% V̇o_2max_ and 30-min moderate-intensity cycling at 50% V̇o_2max_. Rest (5 HR samples) and each exercise intensity (each another 5 HR samples) was conducted once at sea level, and twice at 4,000 m altitude. At each visit to 4,000 m altitude, individuals either performed a workload that corresponds to 35% and 50% sea-level V̇o_2max_ (same absolute workload), or 35% and 50% altitude V̇o_2max_ (same relative workload). The data were reported for every 5 min, thereby totaling 45 heart rate data points per individual across normoxia and hypoxia, as well as across relative and absolute workloads (total samples = 270). For the validation, progressive dehydration level was again approximated by linear interpolation over the exercise period using *Eq. 37* and used in the calculation of the additive CHSI (*Eq. 22*).

The results of the validation indicate a strong relationship (measured HR = 0.76**·**predicted HR + 18.8, *R*^2^ = 0.91) between measured and predicted HR, which was increased further when the data were stratified by altitude and workload type (measured HR = 0.77**·**predicted HR + 17.4; *R*^2^ = 0.99). The validation shows that the model accounts well for the increase in HR induced by ascent to altitude.

## DISCUSSION

Using regression analyses of humans physiological responses to heat, altitude, and dehydrative stress (40, 47, 57), this study proposes a model for predicting cardiovascular responses to environmental stressors, at rest, and during exercise. Independent comparison with empirical data representative of a heterogenous population, performing various exercise intensities, in range of environmental conditions, demonstrated that the CVR Model performs well in predicting cardiovascular strain at an individual and group mean level (Fig. 5).

### Validations 1 and 2: Heart Rate Responses to Heat and at Altitude Exposure

Validation 1 shows that both the synergistic and additive CHSI (*Eq. 22*) provided strong predictions of cardiovascular strain in resting and exercising individuals exposed to heat stress. Nevertheless, the additive CHSI model provided a superior prediction (Fig. 4). This observation perhaps supports our postulation that untrained individuals would be best represented by the additive CHSI, whereas endurance-trained athletes would exhibit an interaction between body temperature and hydration level on cardiac function that is synergistic in nature.

Another observation from the heat exposure validations was that the absolute prediction was an underestimation of HR across all fitness levels (Fig. 5). The most plausible explanation for this is an underestimation of the heat sensitivity coefficients used in *Eqs. 23–26*, which were based on a relatively small number of observations across only two experimental laboratories (47–54), and/or a *y*-intercept deviation in basic HR_rest_ calculation. Indeed, increasing the heat sensitivity coefficients by a factor of 1.5 in *Eqs. 23–26* (to −0.125, −0.125, 0.038, 0.47 in *Eqs. 23–26*, respectively), as well as increasing the HR_rest_*y*-intercept by 10 beats·min^−1^ (*Eq. 9*; HR_rest_ = 100.93 −0.64·V̇o_2max_/mass·1,000) largely eliminates this underestimation. Although minor adjustments will ultimately serve to rectify estimation errors, it is proposed that further validations should be conducted to ascertain the error consistency across data sets. Whether the error is maintained when integrated with an advanced thermophysiological model and/or combined with a continuous measurement of fluid loss during rest and exercise in the heat will be an important question for future validation work.

### Advanced Thermophysiological Models and Impact of the CVR Model

As noted earlier, the present CVR Model would benefit from being combined with advanced thermophysiological models providing appropriate temporal input to which the CVR model can respond. If successfully integrated, such combined models would provide a convenient and inexpensive method for evaluating human cardiac strain under intense physical workloads, and stressful environmental conditions.

A unique aspect of the present CVR model, as well as any future combined CVR-thermophysiological models, is that upper limits on HR, SV, and CO (thereby regional blood flows, and V̇o_2max_) are imposed, therefore allowing an estimation of whether a given exercise load is attainable. As such, utility of future models could be extended to simulate athletes’ physiological responses during running exercise in a range of environmental conditions, as well as the basis for interindividual prediction of work tolerability, occupational safety, and exercise performance modeling.

In addition to an advanced capacity for thermophysiological modeling, the estimation of cardiac specific parameters provides a mathematical means by which complex thermophysiological models can be validated against field-based metrics, such as heart rate—a widely used and highly accessible metric in training and exercise science, as well as the main basis of the popular wristwatch training systems. An additional utility of the present CVR Model is the capacity to provide a more realistic estimate of CO and its limits, and further calculate the proportion of this volume required for regional tissue blood perfusion rates in skin, active muscles, and the major organs during exercise, across different environmental conditions (i.e., *Eqs. 33–35*). This differs from the current method of assuming a predefined requirement for muscle oxygen extraction (i.e., ${\text{AVO}}_{{\text{2}}_{\text{diff}}}$) across exercise intensities, systemic oxygen concentration levels, and thermophysiological states, as proposed in previous thermophysiological models. As a result, the present models’ active muscle oxygen extraction is variable across exercise intensities, systemic oxygen concentration levels, and thermophysiological states (Fig. 4).

### Perspectives and Limitations

It is important to note that metrics such as SV and CO were not directly validated in the present study. However, an accurate prediction of HR is unlikely if indeed CO and SV were not largely well accounted for by the model. Other parameters such as regional blood flow or oxygen extraction, are difficult to validate without access to large, dedicated experimental databases. Given the CVR Model can simulate many thousands of possible combinations of individual, environmental, and workload situations, a complete validation of the complex relations is a major task for future work. To this point, the ongoing work on the implementation of the present CVR Model in the FPC Model (3, 4, 8) should enable a rigorous, in-depth testing of the CVR model in conjunction with an advanced mathematical model of human thermoregulation. It is our hope that the increasing availability of open access data repositories in the physiological sciences will assist in this process.

Another current limitation of the CVR Model is that it does not currently estimate, e.g., time to exhaustion during submaximal work per se, nor a self-paced work completed over a fixed exercise time or distance; and does not account for the acclimation status. To achieve this, future model development will be needed to integrate, e.g., a lactate threshold, long-term environmental exposure time, critical power, V̇o_2_ slow component, muscle fatigue, and/or anaerobic reserves, to estimate performance of an individual per se (62, 63). This may require extending the model with more circulatory parameters (64). However, the present model provides a step forward in the development of an advanced exercise regulation model that could in turn be used to explore our fundamental understanding of the key principles influencing exercise performance in humans (3, 58, 65).

### Conclusions

By estimating cardiac parameters, the present research assists in the application of advanced thermophysiological modeling to the estimation of cardiovascular strain, regional blood flow, and oxygen extraction, across a range of individuals during acute exposure to complex environmental conditions. The development and inclusion of cardiac parameters and limitations provides a basis for thermophysiological modeling of interindividual prediction of work tolerability, occupational safety, and exercise performance modeling, as well as simple-to-validate metrics of cardiovascular function, and a method to evaluate fundamental understanding of the principles influencing exercise and thermoregulation in humans (3, 58, 65).

## GLOSSARY

%Dehyd: Predicted percentage dehydration during exercise

${\text{AVO}}_{{\text{2}}_{\text{diff}}}$

: Basic arterial blood O_2_ extraction (total tissue average)

${\text{AVO}}_{{\text{2}}_{\text{diff(altitude)}}}$

: Arterial blood O_2_ extraction modulated for altitude

${\text{AVO}}_{{\text{2}}_{\text{diffmax}}}$

: Basic maximum arterial blood O_2_ extraction (total tissue average)

${\text{AVO}}_{{\text{2}}_{\text{diffmax(altitude)}}}$

: Maximum arterial blood O_2_ extraction modulated for altitude

${\text{AVO}}_{{\text{2}}_{\text{diffrest}}}$

: AVO_2diffrest_ Resting arterial blood O_2_ extraction (total tissue average)

${\text{AVO}}_{{\text{2}}_{\text{diffrest(altitude)}}}$

: Arterial blood O_2_ extraction modulated for altitude
Basic: Term to describe variables not yet modulated for environmental strains.
CHSI: Cardiac heat strain index
CO: Basic cardiac output
CO_(heat,altitude)_: Basic cardiac output modulated for heat, dehydration, & altitude
CO_(altitude)_: Basic cardiac output modulated for altitude
CO_max_: Basic maximum cardiac output

${\text{CO}}_{{\text{max}}_{\left(\text{heat},\text{altitude}\right)}}$

: Basic maximum cardiac output modulated for heat, dehydration, & altitude
CO_rest_: Basic resting cardiac output

${\text{CO}}_{{\text{rest}}_{\left(\text{heat},\text{altitude}\right)}}$

: Basic resting cardiac output modulated for heat, dehydration, & altitude
CoreBF: Core (major organs and viscera) blood flow
CVR Model: Cardiovascular response Model
FPC Model: Fiala Thermal Physiology & Comfort Model

${\text{F}\dot{\text{V}}\text{O}}_{2{\text{max}}_{\left(\text{reserve}\right)}}$

: Workload as a fraction of the difference between V̇o_2rest_ and V̇o_2max_

${\text{F}\dot{\text{V}}\text{O}}_{2{\text{max}}_{\left(\text{reserve},\text{heat},\text{altitude}\right)}}$

: Workload as a fraction of the difference between V̇o_2rest_ and V̇o_2max_ when corrected for cardiac output changes associated with heat and altitude
HR: Basic heart rate
HR_(heat,altitude)_: Heart rate modulated for heat, dehydration, & altitude
HR_max_: Basic maximum heart rate
HR_rest_: Basic resting heart rate

${\text{HR}}_{{\text{rest}}_{\left(\text{heat},\text{altitude}\right)}\;}$

: Resting heart rate modulated for heat, dehydration, & altitude
MusBF: Active leg muscle blood flow
SkBF: Skin blood flow
SV: Basic stroke volume
SV_(heat,altitude)_: Basic stroke volume modulated for heat, dehydration, & altitude
SV_max_: Basic maximum stroke volume

${\text{SV}}_{{\text{max}}_{\left(\text{heat},\text{altitude}\right)}\;}$

: Basic maximum stroke volume modulated for heat, dehydration, & altitude
SV_rest_: Basic resting stroke volume

${\text{SV}}_{{\text{rest}}_{\left(\text{heat},\text{altitude}\right)}\;}$

: Basic resting stroke volume modulated for heat, dehydration, & altitude
*T*_body_
: Mean body temperature (°C) is calculated as: 0.2 × *T*_skin_ + 0.8 × *T*_core_
*T*_core_
: Core temperature
*T*_skin_
: Mean skin temperature
V̇o_2max_: Basic maximum oxygen consumption

${\dot{\text{V}}\text{O}}_{2{\text{max}}_{\left(\text{heat},\text{altitude}\right)}\;}$

: Basic maximum oxygen consumption modulated for heat, dehydration, & altitude
V̇o_2rest_: Basic resting oxygen consumption

## GRANTS

The research presented was cofunded by the Adidas Sport Science Team, Germany, and the Environmental Ergonomics Research Center, Loughborough University, UK.

## DISCLOSURES

No conflicts of interest, financial or otherwise, are declared by the authors.

## AUTHOR CONTRIBUTIONS

A.L., D.F., C.H., and G.H. conceived and designed research; A.L. performed experiments; A.L., D.F., and G.H. analyzed data; A.L., D.F., C.H., and G.H. interpreted results of experiments; A.L. prepared figures; A.L. drafted manuscript; A.L., D.F., C.H., and G.H. edited and revised manuscript; A.L., D.F., C.H., and G.H. approved final version of manuscript.
